# From risk communication about asymptomatic atherosclerosis to cognitive and emotional reactions and lifestyle modification

**DOI:** 10.1186/s40359-023-01467-x

**Published:** 2024-01-24

**Authors:** Elin M. Andersson, Kristina Lindvall, Patrik Wennberg, Helene Johansson, Steven Nordin

**Affiliations:** 1 Department of Psychology, Umeå University, S-901 87 Umeå, Sweden; 2 Department of Epidemiology and Global Health, Umeå University, Umeå, Sweden; 3 Department of Public Health and Clinical Medicine, Umeå University, Umeå, Sweden

**Keywords:** Atherosclerosis, Lifestyle, Health behaviour, Prevention, Health promotion, Decision making, Cognition, Emotion

## Abstract

**Background:**

Non-adherence in the general population to preventive guidelines on cardiovascular disease calls for an interdisciplinary approach acknowledging psychological factors of relevance for risk communication and lifestyle modification. Evidence is building up regarding the advantage of sharing arterial imaging evidence of subclinical atherosclerosis with asymptomatic individuals, but there is limited understanding of how this relates to mechanisms of importance for behavioural change. Longitudinal studies on associations between patients’ reactions and lifestyle modification are missing. The population-based randomized controlled trial VIPVIZA investigates the impact of pictorial information about subclinical atherosclerosis, added to traditional risk factor-based communication. The intervention includes a personalized, colour-coded and age-related risk communication strategy and a motivational conversation, and has been shown to reduce cardiovascular disease risk.

**Methods:**

In the present study we assessed cognitive and emotional reactions to the intervention, and how these reactions are associated to lifestyle modification. The participants’ evaluation of the risk communication was assessed in the intervention group (*n* = 1749). Lifestyle modification was assessed with a lifestyle index based on physical activity, diet, smoking and alcohol consumption at baseline and after 3 years. Associations between cognitive and emotional response and lifestyle modification were tested with analyses of covariance in a subset of participants (*n* = 714–857).

**Results:**

The intervention increased understanding of personal CVD risk, the possibility to influence the risk, and how to influence the risk. Severity of atherosclerosis was associated with emotional reactions, but emotions of strong negative valence were uncommon. Cognitive response and emotional arousal evoked by the intervention were positively associated with lifestyle modification, whereas negative emotions in isolation were not. High level of cognitive response in combination with high level of emotional arousal was found to be most beneficial for lifestyle modification.

**Conclusions:**

The results demonstrate the potential of communicating asymptomatic atherosclerosis with a pictorial, colour-coded and age-related strategy, also including a motivational conversation. Furthermore, the results show the importance of CVD risk communication evoking engagement, and that an interaction between cognitive and emotional reactions might be central for sustained lifestyle modification. Our results also indicate that, in an asymptomatic population, atherosclerosis screening may strengthen disease prevention and health promotion.

**Trial registration:**

ClinicalTrials.gov identifier: NCT01849575. Registration date 08/05/2013.

**Supplementary Information:**

The online version contains supplementary material available at 10.1186/s40359-023-01467-x.

## Background

There is a great need of effective cardiovascular disease (CVD) prevention targeting lifestyle modification. Prevalence of obesity and type 2 diabetes are increasing across all 56 European Society of Cardiology (ESC) member countries [1], and mortality from CVDs has increased worldwide in the past decade [2]. Promotion of a healthy lifestyle is the most important way to prevent atherosclerotic CVDs [3]. Non-adherence to preventive guidelines and treatment is of great concern [4]. An interdisciplinary approach acknowledging psychological factors of relevance for risk communication and lifestyle modification is important to meet the need of more effective prevention. The ESC has in its latest version of *Guidelines on cardiovascular disease prevention in clinical practice* included a section on communication of cardiovascular risk, and addressed the issue of importance to assess whether patients understand their risk, the anticipated risk reduction, and the pros and cons of an intervention [3]. Studies assessing the effect of using cardiovascular risk scores seldom investigate whether interventions improve the participants’ ability to report an estimate of their personal risk [5]. Furthermore, improved accuracy of perceived risk is not equivalent to the risk being considered as relevant, emotionally engaging or useful for health-oriented decision-making.

Numerically expressed probability of risk is cognitively challenging, and can easily lead to misinterpretations [6, 7]. In contrast, pictorial health information can improve knowledge and understanding, especially in persons with low health literacy [8]. However, pictorial components vary considerably between studies, and it is unclear as to what constitutes an optimal format. Graphical material, film, pictures, even raw medical images can be used, and the additional explanations provided can be any combination of verbal communication, numerical risk assessment or written information. Furthermore, risk communication varies on dimensions such as risk for disease and/or death; conceptualization of disease process; time horizon; personalized, general or hypothetical risk; visualization of quantitative aspects such as numerical risk estimates or qualitative aspects in terms of high or low risk, or risk related to or expressed as age [8–13]. Presentation of absolute, rather than relative, risk reduction is more effective [14], and presentation of lifetime risk for CVD, rather than 10-year risk, has been shown to result in respondents rating the risk as higher [15].

Visualization of numerical risk estimates rather than qualitative aspects of risk is common [16], but can be misleading, for example, when modest risks are presented as proportions. It is therefore important to test not only patient satisfaction and understanding of risk formats, but also how it influences adherence to medication and lifestyle modification [15]. The use of medical imaging technologies is increasing, and it has been shown that feedback of medical images to individuals has the potential to motivate risk-reducing behaviours and reduce risk factors [17]. Health risk needs to be communicated and presented in a format that gives *meaning* to the person, so that expression of risk leads to qualitative experiences of the risk being high or low, and where emotions are acknowledged for shaping mental representations of risk [18]. Concepts such as “heart age”, “vascular age” and “risk age” link individual risk factors to life years in a way that may be intuitively and personally engaging [19]. Age-based strategies of CVD risk communication are promising, and vivid risk presentations are thought to promote behaviour change. However, the available evidence is sparse, and strategies differ significantly regarding central aspects, calling for further studies [9, 20]. Longitudinal studies are also needed since interventions for chronic conditions, including visual elements, often assess the impact on illness beliefs and adherence after short follow-up durations [10].

Patients prefer personalized risk information over standard risk factor material [21], but a systematic review of systematic reviews found that the support for personalized risk communication as a way to motivate lifestyle modification is weak. However, pictorial techniques were found to be the most promising method of communicating individual risk [11].

Early models predicting health behaviour had a strong cognitive focus [22], whereas more recent models acknowledge the relevance of emotions [23, 24]. An individual’s capability, opportunity and motivation for health [25] and complex relations between cognition and affect as well as contextual factors [26] are also acknowledged.

Evidence is building up regarding the advantage of sharing arterial imaging evidence of subclinical atherosclerosis with asymptomatic individuals as a way of improving risk control and clinical outcomes [27]. Atherosclerosis screening with ultrasound, which is non-invasive and cost effective, and therefore enables screening of populations [28, 29], can be used as part of CVD risk communication [30]. Visualization of the underlying process of CVD can give patients an experience of “direct evidence” of disease, clarify personal risk and thereby increase engagement.

Fortunately, the development of atherosclerosis can be slowed down and even reversed [31]. Ultrasound techniques enable examination of CVD risk by assessment of atherosclerosis while still asymptomatic, but evidence for its contribution to lifestyle modification is inconsistent. Visual feedback by means of individual medical imaging results has been found to change behaviour regarding smoking cessation, whereas trials reporting physical activity, dietary intake and medication adherence have shown no significant effects [12]. A systematic review assessing atherosclerosis screening found improvement on several outcomes such as risk perception, diet, motivation and blood lipids [13]. Carotid ultrasound screening can have an impact also on physicians’ risk perception, support patient-centred consultation, and improve shared decision-making [32] and CVD risk management [33, 34]. However, there is a knowledge gap in the literature regarding assessments of participants’ appraisals and perceptions of cardiovascular imaging interventions, according to a systematic narrative review of the impact of vascular screening interventions on perceived susceptibility to CVD, efficacy beliefs and behavioural intentions [35]. Furthermore, studies assessing reactions and behaviours short in time after an intervention [36, 37] have advantages regarding the possibility to study the association between immediate reactions and initiation of behaviours, however, such studies do not address the question whether initial reactions or actions transfer to long term behavioural change and a sustainable lifestyle.

The present study was based on data from the Visualization of Asymptomatic Atherosclerotic Disease for Optimum Cardiovascular Prevention (VIPVIZA) trial. The pragmatic randomised controlled trial VIPVIZA provides evidence of the contributory role of pictorial presentation of atherosclerosis for reduction of CVD risk factors, even with a sustained effect over three years, and regardless of participants’ education level [30, 38]. The primary study at 3-year follow up showed that for the intervention group, who received personalized, colour-coded and age-related information about atherosclerosis status, Framingham Risk Score was 13.38, whereas it was 14.08 for the control group (*p* = 0.047) and SCORE was 1.69 vs. 1.82 (*p* = 0.022) [38]. A secondary analysis, assessing how participants in the intervention group perceived the intervention, and whether their reactions to the intervention were related to long-term behavioural change, is therefore motivated.

A pragmatic RCT is typically characterized by addressing unarguably important outcomes such as mortality and morbidity, inclusion of a wide range of participants and applicability to usual care settings, with the purpose to inform decisions about practice [39]. In line with this, we aimed at contributing with knowledge about how this specific innovative intervention affects the participants, which in turn can contribute to understanding why the intervention is effective, and thereby possible inform intervention design within cardiovascular disease prevention in general and atherosclerosis screening in particular.

The following research questions were addressed in this study: (i) How is personalized pictorial risk communication on atherosclerosis perceived regarding comprehension, contribution of the pictorial information to understanding CVD risk, and contribution of a follow-up nurse phone call for the understanding of the information? (ii) What are the cognitive responses to pictorial risk communication and the follow-up phone-call, do these depend on the level of education, and what are the emotional responses to pictorial risk communication? (iii) To what extent do the cognitive and emotional responses correspond to severity of atherosclerosis presented by the risk message? (iv) Are the cognitive/emotional responses to the pictorial risk communication and follow-up phone call associated with lifestyle modification over three years?

## Method

### Participants

The present study includes only participants in the intervention group of the VIPVIZA trial. For natural reasons, reactions to the intervention is not possible to assess in the control group, since they did not receive the intervention. Of the 1749 participants in the intervention group, 1397 participated in the current study since questions regarding the participants’ assessment of and response to the intervention were not included until a few months into the 3-year follow up.

### Study context and design of the VIPVIZA trial

VIPVIZA, conducted in Västerbotten county in northern Sweden, is a pragmatic, open-label, randomized controlled trial with masked evaluators (PROBE) that investigates the impact of pictorial information about subclinical atherosclerosis, added to traditional risk factor-based communication. VIPVIZA is integrated in primary care via the Västerbotten Intervention Program (VIP), which offers screening for CVD risk factors and individual health promotion counselling to all inhabitants of the county the year they turn 40, 50, and 60 years (*n* = 6,500–7,000 per year) [40]. Participation rate for VIP during the inclusion period was 68%, and only small social selection bias has been observed [41].

For VIP participants aged 60 years, age alone constitutes inclusion criteria (64% of the VIPVIZA study population), those aged 50 years were included due to at least one conventional CVD risk factor (28%), and those aged 40 years on the bases of history of early CVD among first-grade relatives (8%). This means that also individuals with low to moderate CVD risk were included, and that the atherosclerotic disease, when present, was asymptomatic. These features enhance generalization to the general population, rather than to a clinical CVD population. Recruitment was done in different parts of the county during three years, aiming to obtain a representative sample, large enough according to a power-estimation, and facilitated by only one team travelling across the county to perform the ultrasound examinations. In total, 4,177 VIP participants were invited to the VIPVIZA study, and participation rate was 84.6% (*n* = 3532). Participants were included in the study during April 2013-June 2016, and were consecutively and randomly assigned 1:1 to the intervention (*n* = 1749) or the control group (*n* = 1783). For an overview of the study, including intervention components and behaviour change techniques described in terms of the Behaviour Change Technique Ontology, [42] see Additional file 1. Furthermore, a source file of relevance for the ontology is provided in Additional file 2.

In the VIP, all participants responded to a questionnaire covering life style, medication, psychosocial situation, and family history of CVD and diabetes. Blood pressure and anthropometric measurements were taken, and blood samples were collected to measure lipids and blood sugar. At the baseline visit in VIPVIZA, participants also responded to several validated questionnaire instruments, e.g. on health literacy, coping, self-efficacy and depression/anxiety. Presence of carotid atherosclerotic plaque and intima media thickness (IMT) was assessed with ultrasound. Examinations were performed by sonographers specially trained in carotid ultrasound techniques with a mobile *CardioHealth Station*, provided by Panasonic Healthcare Corporation of North America, Newark, NJ, USA. VIPVIZA applies the Mannheim consensus definition of carotid artery plaque. After the baseline visit, the intervention group and their primary care physicians received a letter with the pictorial presentation of the ultrasound result. Also, within 2–4 weeks after they had received the letter, the participants in the intervention group were contacted by a research nurse by telephone for clarifications if needed, any remaining questions and a motivational interview [43] (referred to as the nurse follow-up call). In order to evaluate the effect of the intervention, the control group and their primary care physicians did not receive the result from the baseline ultrasound. After six months, participants in the intervention group once again received the letter with the pictorial presentation of the ultrasound result, including also a reminder of preventive measures. After nine months, and also after 2 and 2.5 years, participants in the intervention group received letters reminding them about the next follow-up visit. These letters contained general information about proceedings in the study and the importance of a healthy lifestyle to prevent progression of atherosclerosis, but no personalized health information was given in the letters. No information letters were sent to the control group.

At 1-year follow up, clinical risk factors were measured again, and all participants and their primary care physician were given the results. Participants also responded to a shorter questionnaire on preventive medication and life style. At 3-year follow-up, the participants were again examined with ultrasound and the full baseline routine of validated psychometric questionnaires, blood samples and measurements of anthropometric data and blood pressure. In addition to this, the intervention group also responded to questions on cognitive and emotional reactions to their first ultrasound result letter, and the nurse follow-up call. These reactions were analysed in the present study. After this visit, all participants received a letter with pictorial presentation of their ultrasound result. The complete study protocol is available at https://clinicaltrials.gov/ct2/show/NCT01849575.

#### Pictorial risk communication and motivational interview as intervention

**Fig. 1:**
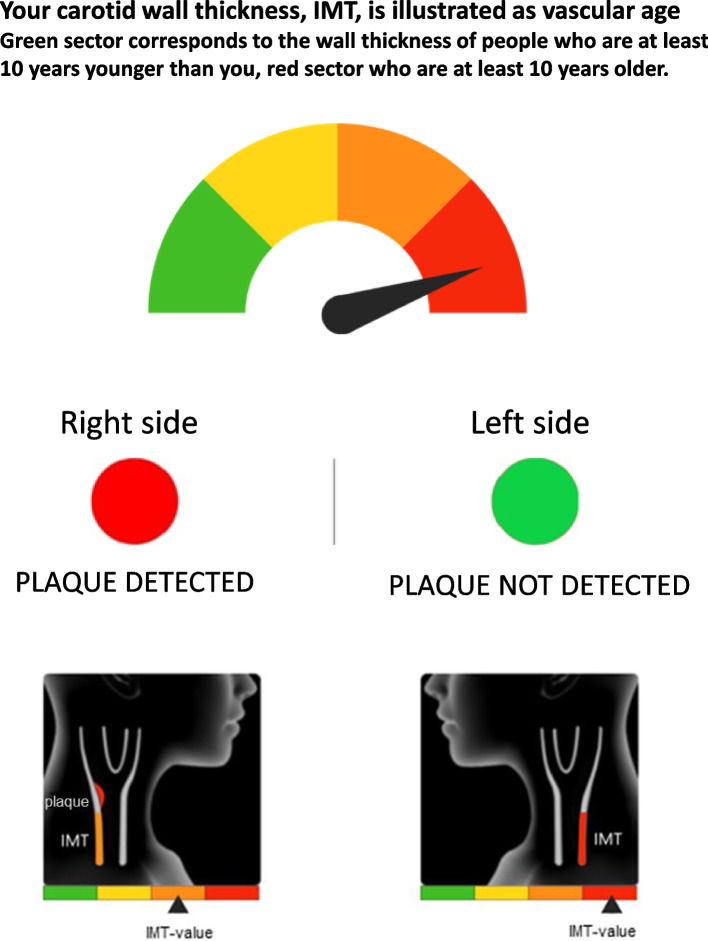
Illustration of graphical elements in the result letter

IMT was communicated as vascular age. Here the individual’s IMT was compared to that of individuals with the same sex and age in a reference population [30], and depicted as a graphical continuous gauge ranging from green via yellow and orange to red. Green corresponds to the IMT of a person at least ten years younger, and red corresponds to an IMT of a person being at least ten years older than the participant’s actual age. Plaque was presented as a traffic light with a red (plaque identified) or green (no plaque) dot. An illustration of graphical elements in the letter is provided in Fig. 1. Written information was also provided in the same letter, describing atherosclerosis as a dynamic process that can be slowed or even reversed by healthier life style and preventive medication. Within 2–4 weeks after participants in the intervention group had received the letter, they were contacted by a research nurse by telephone for clarifications if needed, any remaining questions and a motivational interview. The latter is a method for collaborative communication aiming to enhance readiness for change, where evoking the client´s own perceptions, values and motivations for change is central [43].

### Ethical considerations

All study participants provided written informed consent when included in the VIPVIZA study. The present study was approved by the Umeå Regional Ethics Board (2011–455-31 M and 2012–463-32 M).

### Material

Data on the participants’ perceptions of and reactions to the intervention was collected at the 3-year follow-up, and data on health behaviours was collected at baseline and 3-year follow-up.

#### Participants’ assessment of the intervention

Comprehension of the ultrasound result letter was assessed with the question *How would you assess your understanding of the letter?* Five response alternatives were given: *It was very easy, It was rather easy, It was rather difficult, It was very difficult,* and *Do not remember/know.* Assessment of the contribution of the ultrasound result letter for understanding personal CVD risk was assessed with the question *Did the letter contribute to you understanding your risk of cardiovascular disease better than before?* (1 missing case, out of 1397). Assessment of the contribution of the nurse phone call for understanding of the ultrasound result letter was assessed with the question *Did the follow-up call contribute to understanding the letter?* Response alternatives for these two questions were *Very much, Rather much, To some extent, Not at all, Do not remember/know* (25 missing cases).

#### Cognitive impact of the intervention

One question assessed risk perception and two questions assessed efficacy beliefs. For each statement, participants were asked to evaluate to what extent the letter and the phone call in combination had contributed: *The letter and the phone call in combination have contributed to that: (i) … I better understand my personal risk for cardiovascular disease* (43 missing). *(ii) … I better understand my possibility to influence my risk* (40 missing)*. (iii) … I better understand how I can influence my risk* (44 missing)*.* For each statement, the alternatives were *Completely agree, Partly agree, Partly disagree, Completely disagree,* and* Do not remember/know.*

For each of the three questions assessing the cognitive impact of the intervention, the response alternative *Completely agree* corresponded to 5 points, *Partly agree* to 4 points, *Partly disagree* to 3 points, *Completely disagree* to 2 points, and *Do not remember/know* to 1 point. When combined, a score of 1–4 was categorized as *Low cognitive response* (*n* = 317), 4.33–4.66 as *Moderate cognitive response* (*n* = 207), and 5 (corresponding to completely agreeing on all three questions) as *High cognitive response* (*n* = 340).

#### Emotional impact of the ultrasound result letter

The participants were asked about their reactions to the ultrasound result letter with the following statements: *I was positively surprised* (54 missing)*; I was relieved/calmed* (52 missing)*; I was worried/afraid* (45 missing)*;* and *I was shocked* (50 missing*.* The response alternatives were *Completely agree, Partly agree, Partly disagree, Completely disagree,* and *Do not remember/know*. For analyses of associations between emotional reactions and lifestyle modification, emotions were assessed as arousal and negative valence (see Statistical analysis section).

When emotional reactions were studied from the perspective of level of negative emotions, participants who replied *Do not remember/know* were excluded from the analyses, since it is not possible to interpret direction of valence from this statement. For each of the questions, the response alternative *Completely agree* corresponded to 1 point, *Partly agree* and *Partly disagree* to 2 points, and *Completely disagree* to 3 points. When the questions were combined, a score of 1–1.5 was categorized as *Low negative emotion* (*n* = 124), 2–2.5 as *Moderate negative emotion* (*n* = 378), and 3 as *High negative emotion* (*n* = 218)*.*

When emotional reactions were studied from the perspective of level of arousal, participants who replied *Do not remember/know* were included in the analyses, since it can be argued that this statement strongly indicates an absence of increased arousal. The response *Do not remember/know* corresponded to 1 point, *Partly agree* and *Partly disagree* to 2 points, and *Completely agree* and *Completely disagree* to 3 points. A score of 1–1.5 was categorized as *Low arousal* (*n* = 125), 2–2.5 as *Moderate arousal* (*n* = 458), and 3 as *High arousal* (*n* = 276)*.*

#### Lifestyle index

The use of lifestyle indices for assessing and predicting CVD risk has previously been described [44]. In VIPVIZA, the lifestyle index is based on the four frequently evaluated health behaviours: physical activity, diet, smoking and alcohol consumption. For each behaviour, a score between 1 and 3 is given, where 1 corresponds to the unhealthiest level and 3 to the most health-promoting level (total score can vary from 4 to 12). Lifestyle modification over three years was derived by subtracting the value of lifestyle index at baseline from that at 3-year follow-up. The definition of levels for physical activity, smoking and alcohol consumption was based on commonly used definitions and clinical guidelines. The measure for diet, described below, was included at baseline, but not at the start of the 3-year follow up, resulting in missing values on lifestyle index. In our sample, 886 individuals provided data on change in lifestyle index (511 missing).

##### Physical activity

Three levels of physical activity were constructed based on assessment of level of physical activity in every-day life and leisure time. These were (1) sedentary, (2) moderate physical activity of < 150 min/week, and (3) around or above the recommended level of physical activity of at least moderate intensity at least 150 min/week.

##### Diet

Diet was assessed with the Food Frequency Questionnaire (FFQ), [45] from which a Healthy Diet Score (HDS) was calculated, reflecting daily intake of eight categories of food and beverages. HDS has been described elsewhere [46], and change in HDS is associated with change in cardio-metabolic risk factors [47]. Fish, fruits, vegetables and whole grains represented favourable food, whereas red or processed meats, desserts and sweets, sugar-sweetened beverages and fried potatoes represented unfavourable food/beverage. For both sexes, intake frequencies were ranked in ascending quartile ranks for favourable foods/beverage groups, and in descending quartile ranks for unfavourable foods/beverage groups. The HDS, ranging from 0 to 24, represents the sum of all quartile ranks where higher rank indicates healthier food and beverage choice. For the Lifestyle index, the three levels of HDS represented (1) the first, (2) second, and (3) third tertile.

##### Smoking

The three levels were (1) Daily smoker, (2) Occasional smoker, (3) Non-smoker.

##### Alcohol consumption

In accordance with the cut-off levels for the Alcohol Use Disorders Identification Test (AUDIT) [48], the three levels were (1) not at risk, (2) harmful use, and (3) abuse or dependence of alcohol.

### Statistical analysis

Chi-square test was used to study associations between cognitive response and level of education as well as severity of atherosclerosis as presented by the risk message and cognitive/emotional reactions. Lifestyle modification over three years was derived by subtracting the value of lifestyle index at baseline from that at 3-year follow-up. The association between lifestyle modification, on the one hand, and cognitive and emotional response, on the other hand, were studied with analysis of covariance (ANCOVA), in which Lifestyle index at baseline was used as covariate. Among the 886 participants who provided data on change in lifestyle index, 864 provided data on the composite variable for cognitive response, and 859 on the composite variable for emotional response.

For the analyses of emotional reactions, only the statements regarding being positively surprised and relieved/calmed was used. The statements regarding being worried/afraid and shocked were excluded since very few participants reported that they to a large extent experienced these reactions, and because the questions used captured emotions of both positive and negative valence.

The impact of the combination of cognitive response and emotional arousal on change in lifestyle index was analysed based on the groups formed in each composite variable, respectively (described under “Material” section). These combinations were low cognition/low arousal (*n* = 275), low cognition/high arousal (*n* = 237), high cognition/low arousal (*n* = 141), and high cognition/high arousal (*n* = 190).

## Results

Demographics and health status at baseline for the 1397 participants are presented in Table 1. The 511 participants who did not provide data on lifestyle index did not differ notably from those providing such data regarding demographics, CVD risk factors, assessment of the intervention or cognitive or emotional reactions.

**Table 1:** Description of the participants regarding demographics and health status at baseline

	Men	Missing	Women	Missing
	(*n* = 641)		(*n* = 756)	
Age, years, n (%)				
40	50 (7.8)		53 (7.0)	
50	171 (26.7)		217 (28.7)	
60	420 (65.5)		486 (64.3)	
Education, n (%)		6		7
Basic or middle	466 (73.4)		454 (60.6)	
High (University)	169 (26.6)		295 (39.4)	
BMI categories, n (%)				1
Normal weight	149 (23.2)		289 (38.3)	
Overweight	324 (50.5)		285 (37.7)	
Obesity	168 (26.2)		181 (24.0)	
Plaque status, n (%)				1
No plaque	320 (49.9)		461 (61.1)	
Plaque	321 (50.1)		294 (38.9)	
IMT color code/plaque, n (%)				1
Green/Yellow, no plaque	105 (16.4)		137 (18.1)	
Orange/Red, no plaque	215 (33.5)		324 (42.9)	
Green/Yellow, with plaque	88 (13.7)		52 (6.9)	
Orange/red, with plaque	233 (36.3)		242 (32.1)	
Systolic blood pressure, mmHg, mean (SD)	132.5 (16.1)	1	127.5 (16.5)	
LDL-cholesterol, mmol/l, mean (SD)	3.51 (0.99)	19	3.56 (0.95)	6
Hypertension medication, n (%)	210 (34.1)	26	220 (30.0)	23
Lipid lowering drug, n (%)	91 (14.8)	26	71 (9.7)	23
Framingham risk score, n (%)		15		10
Low/light risk	111 (17.7)		525 (70.4)	
Moderate risk	274 (43.8)		193 (25.9)	
High/very high risk	241 (38.5)		28 (3.8)	
Perceived health past year, n (%)		2		2
Good	478 (74.8)		536 (71.1)	
Poor	161 (25.2)		218 (28.5)	

**Fig. 2:**
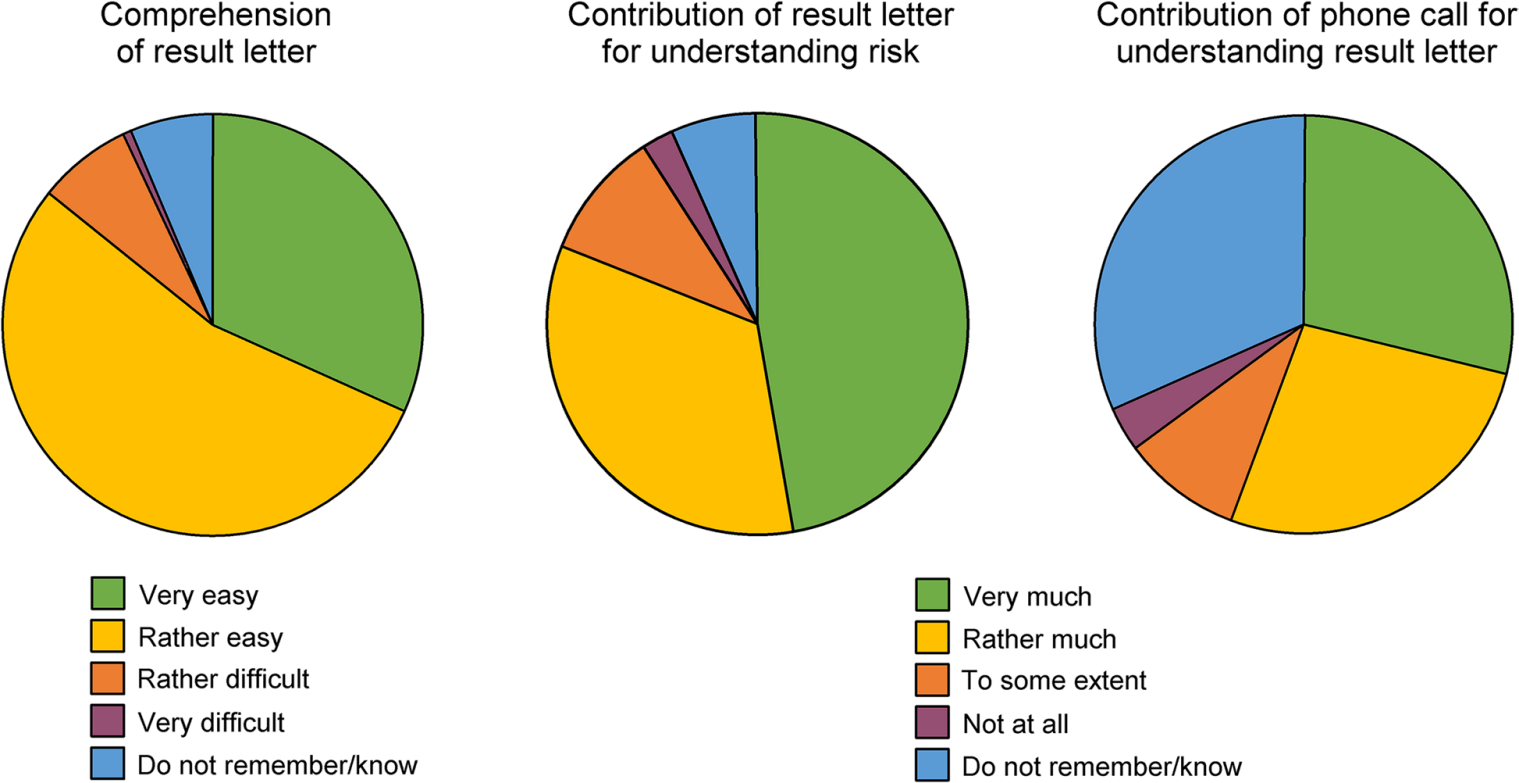
Proportions of reported assessment of the intervention

Results on the assessment of the intervention are presented in Fig. 2. Regarding comprehension of the result letter, 85.8% reported that it was very easy or rather easy to understand the result letter, and 81.1% reported that the result letter contributed very much or rather much to improved understanding of personal CVD risk. Of the sample, 55.0% agreed very much or rather much that the phone call had contributed to understanding of the result letter, but notably, 31.8% replied *Do not remember/know.*

**Fig. 3:**
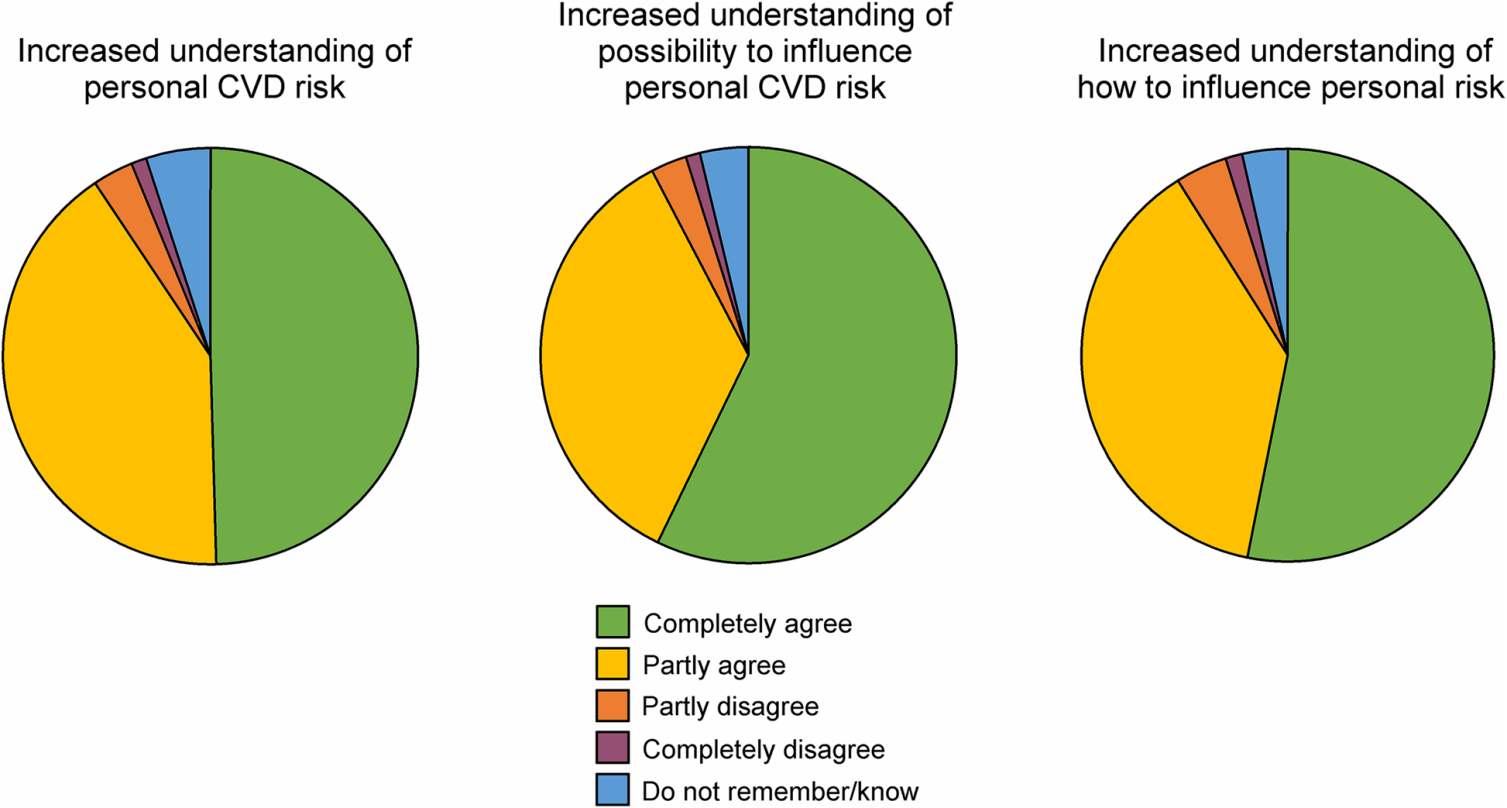
Proportions of reported cognitive impact of the intervention

Cognitive reactions to the intervention are presented in Fig. 3. The intervention had to a large extent contributed to increased understanding of personal CVD risk, the possibility to influence the risk, and how to influence the risk. Furthermore, when assessed with the composite variable, the cognitive impact of the intervention was more commonly reported as high in participants without high education (44.9%) compared to those with high education (30.1%; *p* =  < 0.001).

**Fig. 4:**
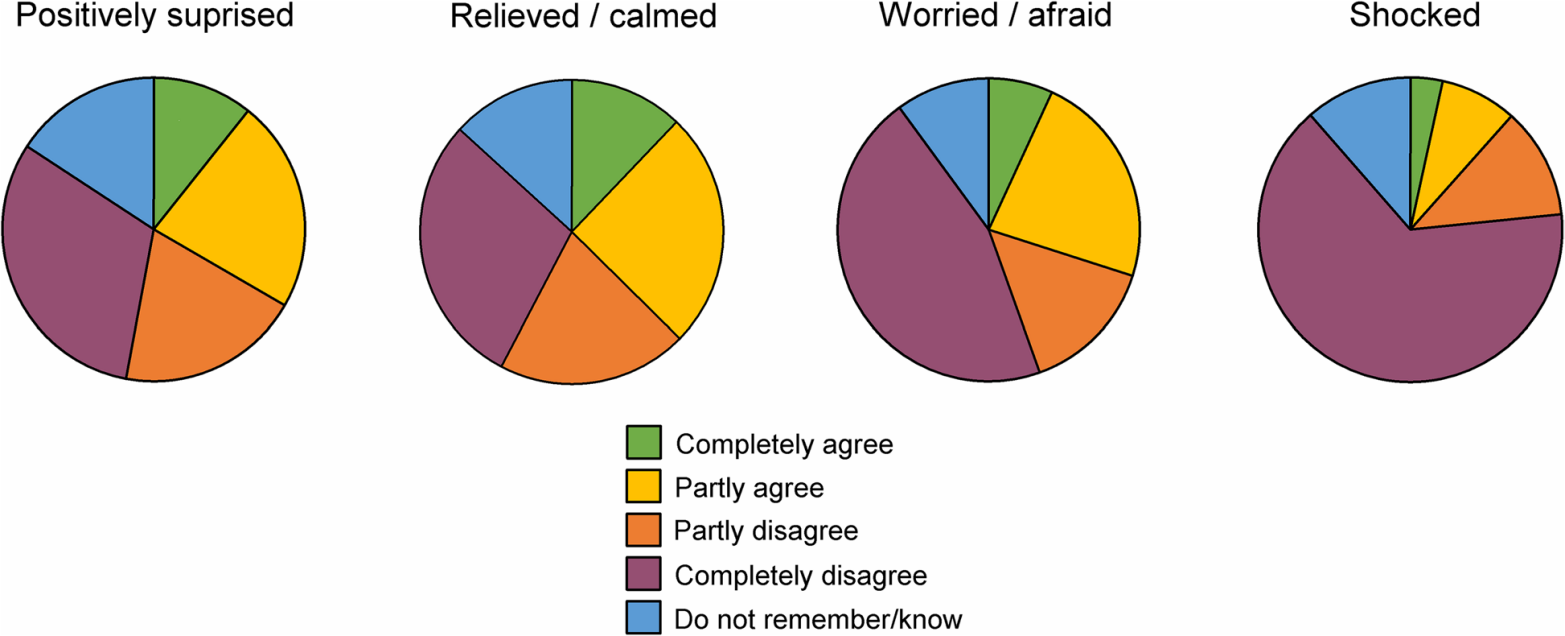
Proportions of reported emotional impact of the ultrasound result letter

Emotional reactions to the ultrasound result letter are presented in Fig. 4. Overall, emotions of strong negative valence, feeling worried/afraid or shocked to a high extent were uncommon. Still, negative emotions and high arousal, assessed with the composite variables, where more common among participants with plaque and orange or red colour code for IMT. Among participants with plaque and orange or red IMT, 48.8% were high in negative emotions on the composite variable. Corresponding proportion was 1.5% for participants without plaque and green or yellow IMT, 29.4% for those without plaque and orange or red IMT, and 30.6% for those with plaque and green or yellow IMT (*p* =  < 0.001).

**Fig. 5:**
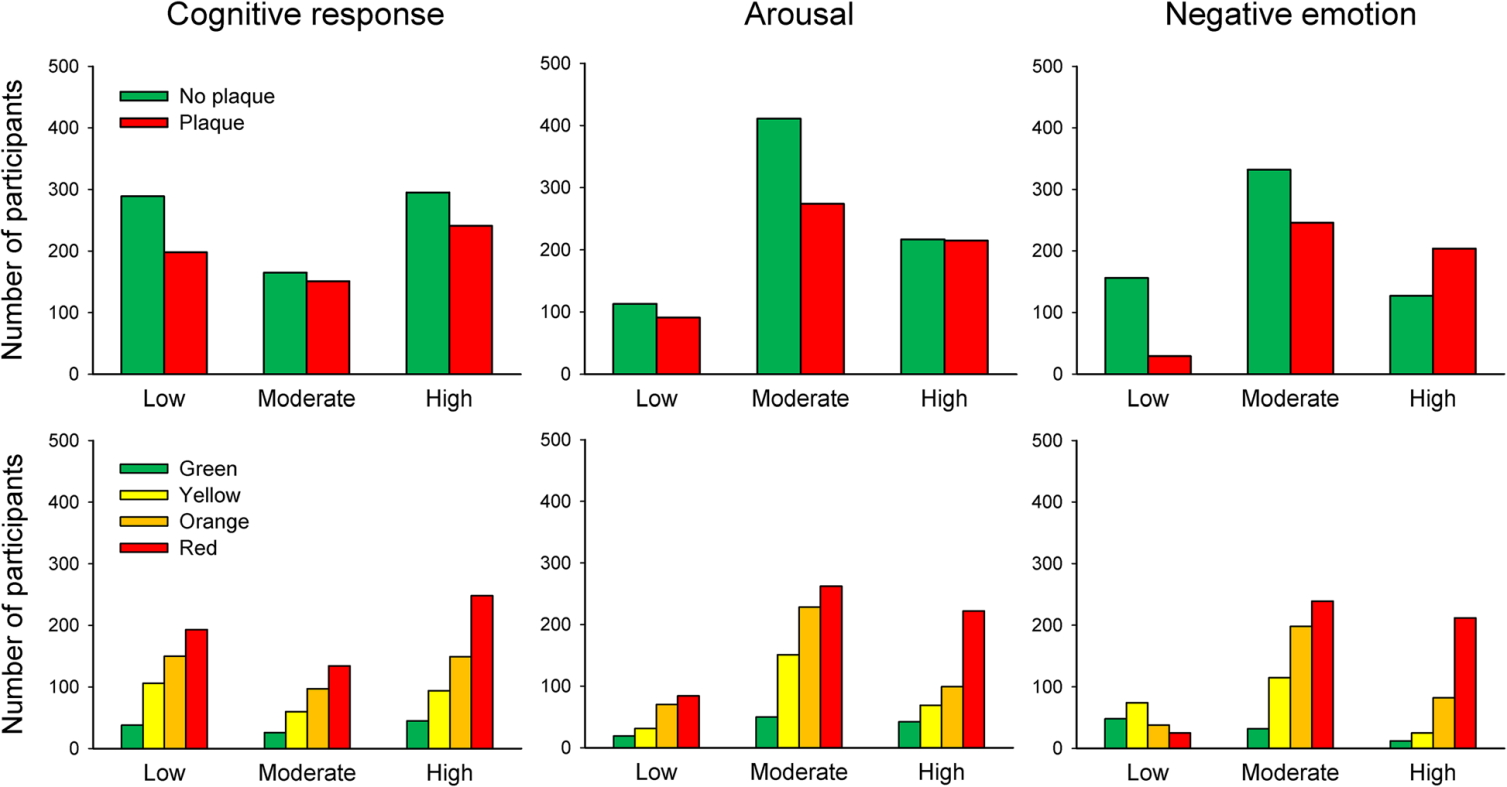
Number of participants reporting various reactions to pictorial information about plaque (upper row) and intima media thickness (IMT; lower row). The IMT colour code is relative to age, such that green corresponds to the IMT of a person at least ten years younger than the participant’s actual age, and red corresponds to an IMT of a person at least ten years older. Cognitive response: plaque *n* = 1339, IMT *n* = 1340. Arousal: plaque *n* = 1321, IMT *n* = 1327. Negative emotion: plaque *n* = 1094, IMT *n* = 1100

Associations between level of reactions and pictorial information about plaque and IMT, separately, are presented in Fig. 5. Chi-square tests regarding plaque/IMT and cognitive response did not reach statistical significance, but so did associations between plaque and arousal (*p* =  < 0.007), plaque and negative emotions (*p* =  < 0.001), IMT and negative emotions (*p* =  < 0.001) and IMT and arousal (*p* =  < 0.001).

**Fig. 6:**
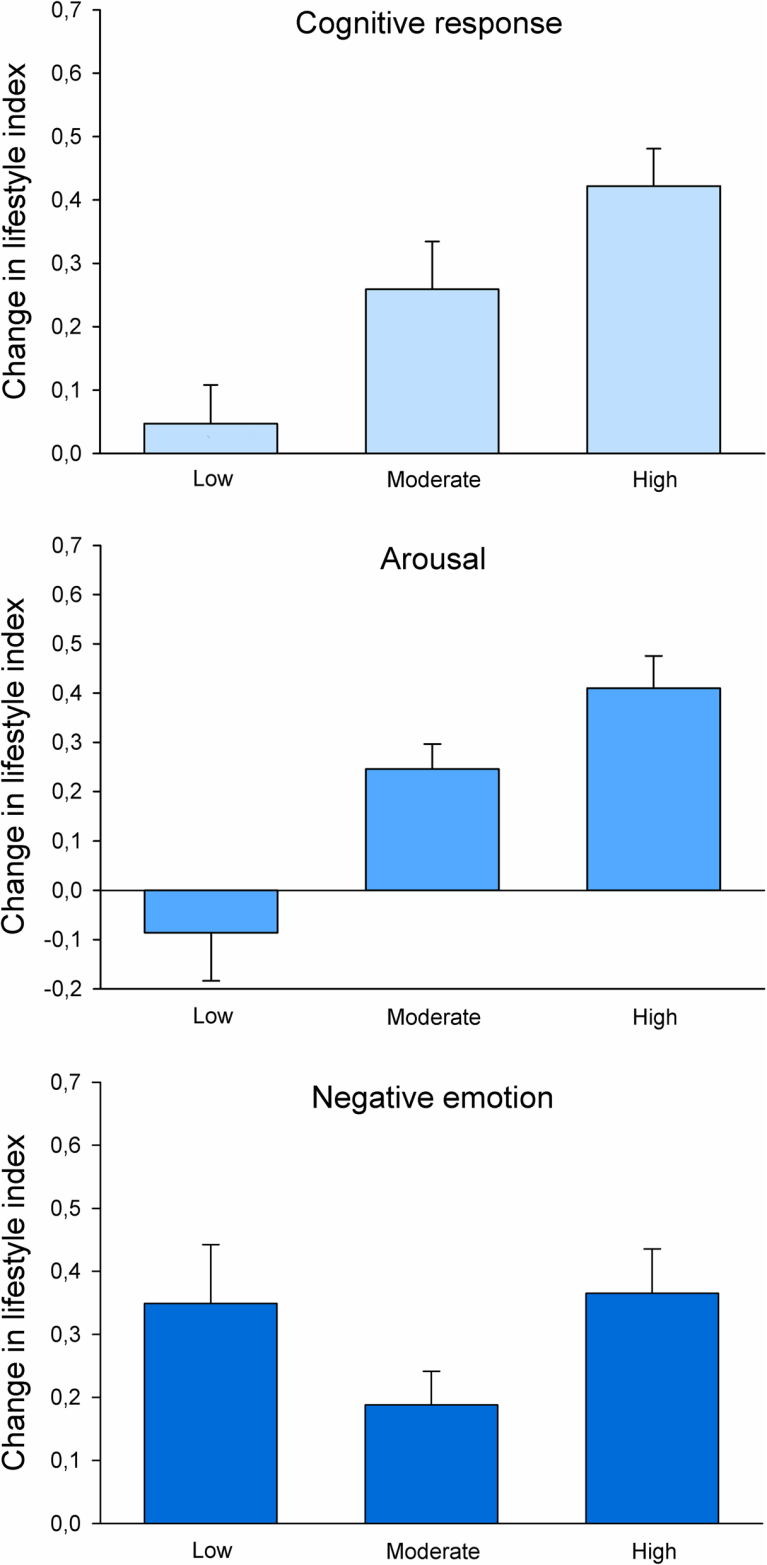
Mean + SE change in lifestyle index as a function of extent of cognitive and emotional reactions on the intervention

The distribution of change in lifestyle index between baseline and 3-year follow-up was approximately normally distributed (range = -3 to 4, mean = 0.253, SD = 1.231), which also applies to the distribution of lifestyle index at baseline. The associations between, on one hand, levels of cognitive response, arousal and negative emotion, and, on the other hand, change in lifestyle index are presented in Fig. 6. There were significant effects of level of both cognitive response and arousal on change in lifestyle index (*p* =  < 0.001), whereas the effect of negative emotion level was non-significant (*p* = 0.089).

**Fig. 7:**
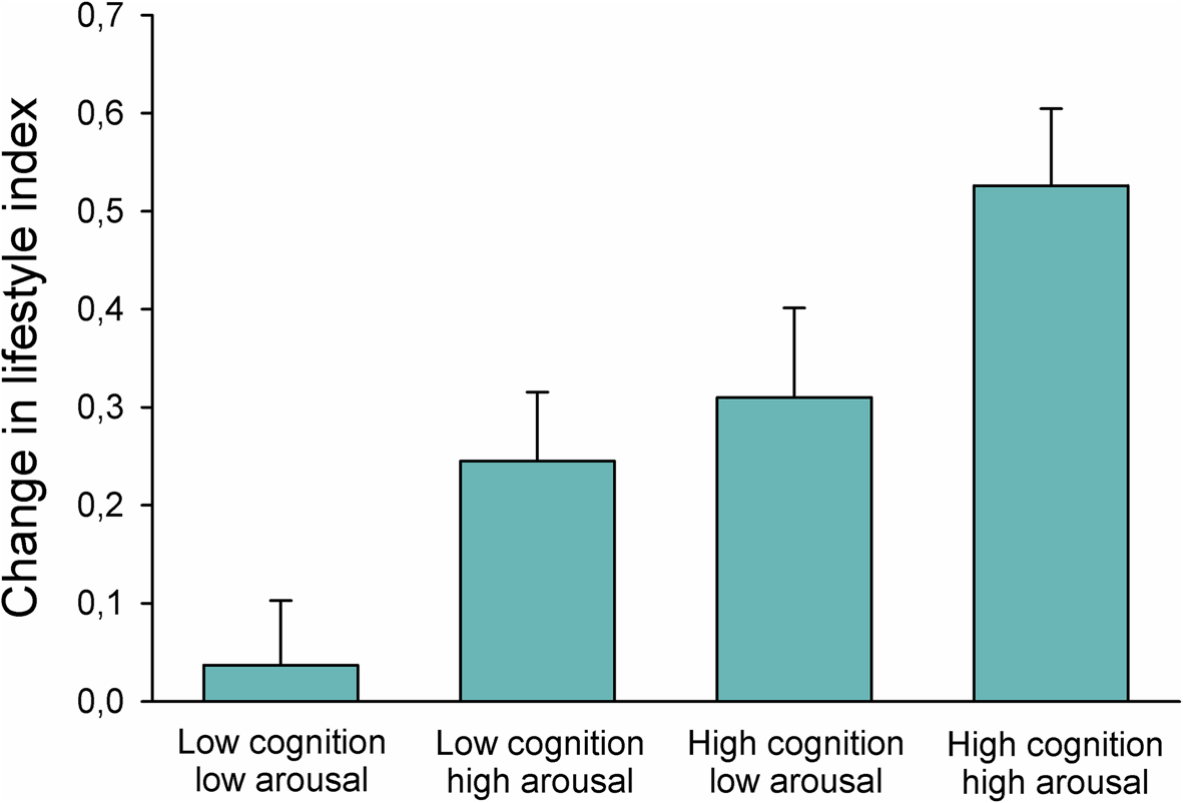
Mean + SE change in lifestyle index in participants with different combinations of low and high cognitive and arousal reactions on the intervention

The association between the combination of cognitive and arousal response level and change in lifestyle index is presented in Fig. 7. There was a significant effect of cognitive response and arousal on change in lifestyle index (*p* =  < 0.001), in which the combination of high level of cognitive response and high level of arousal was found to be most beneficial for lifestyle modification.

## Discussion

To the best of our knowledge, this is the first longitudinal study on associations between reactions on pictorial presentation of atherosclerosis and lifestyle modification. Our findings suggest that cognitive and emotional intervention response, especially in combination, are important for long-term behavioural change. The results are in line with a systematic review on cardiovascular risk communication strategies in primary prevention, which found that strategies that employ personalized and visual evidence of current cardiovascular health status were more likely to promote action to reduce risk [49]. The fact that VIPVIZA is a pragmatic RCT enhances generalization to the general population, rather than to a clinical CVD population, meaning that our findings have the potential to inform intervention design in primary prevention of cardiovascular disease in general and atherosclerosis screening in particular.

In total, 44.1% of the participants had plaque, but, overall, emotions of strong negative valence were not common. Cognitive factors and emotional arousal evoked by the intervention was positively associated with lifestyle modification, whereas negative valence was not. The combination of high level of cognitive response and high level of emotional arousal was found to be most beneficial for lifestyle modification. The pictorial communication conceptualized CVD risk which might be of importance for understanding the link between lifestyle and atherosclerosis development. Furthermore, relating IMT to age and using a color-coded strategy also demonstrated consequences of atherosclerosis. The study supports the importance of interactions between cognitive and emotional factors for sustained adherence to guidelines. Furthermore, our results indicate that, in an asymptomatic population, atherosclerosis screening may strengthen disease prevention as well as health promotion. The overall high level of comprehension of the result letter, and its strong contribution to increased understanding of personal CVD risk are key elements for assessment of reactions and their associations to lifestyle modification. This is in line with a qualitative study in which we assessed reactions evoked by the VIPVIZA intervention and attitudes to lifestyle modifications in participants with improved health status, where the risk message was described as clear, accurate and reliable. The risk message was perceived as convincing and definite in nature, and several participants used the expression “it is stated in black and white…” [50].

Not all participants were reached by the follow-up call, which might explain why more than a quarter did not remember or know whether the follow-up call contributed to understanding the letter. Still, the follow-up call seems to have been important for some participants, which is in line with the findings of a review assessing the effect of visual interventions on illness beliefs and medication adherence for chronic conditions. It showed that interventions using visual elements to conceptualize a condition was most effective if also including human interaction as part of message delivery [10].

The very combination of risk perception and efficacy beliefs is key for successful lifestyle modification. It is therefore promising that the participants to a large extent perceived that the intervention contributed to understanding their individual CVD risk, and to increased understanding of the possibility to influence the risk, and how to do so. The fact that the participants’ assessments are similar across all three aspects is essential also from an ethical point of view, since increased awareness of a health risk can be stressful if there is no possibility to influence the risk.

Fifty percent of the men and 39% of the women received information that they had plaque, and about 45% of the men and 42% of the women received red as colour code for the IMT value, corresponding to a person at least ten years older than the participant’s actual age. However, overall, emotions of strong negative valence were not common. One possible explanation for this is that written information was also provided in the result letter, describing atherosclerosis as a dynamic process that can be slowed or even reversed by healthier life style and preventive medication. Participation in the VIP may also have led to pre-understanding about health status, and thereby influenced expectations. Still, when associations between reactions and risk message were assessed with a variable combining the plaque and IMT results, negative emotions and arousal were more common in the group with plaque and orange or red colour code for IMT.

Associations between reactions and pictorial information were also assessed separately for plaque and IMT value. From the perspective of risk communication, it seems that not only information on plaque, but also on IMT presented as vascular age in colour communicating severity can evoke cognitive and emotional reactions. This is in line with previous research on age-based CVD risk communication strategies [9, 19, 20]. Importantly, in the VIPVIZA study, vascular age is color-coded such that red signals high risk, which may have contributed to communicating severity and susceptibility. Results of our aforementioned qualitative study also showed that plaque as well as vascular age elicited emotional responses. Thus, the interpretation "older than I actually am" may sting [50].

The fact that cognitive and emotional reactions to the intervention transferred into sustained lifestyle modification over three years shows how pictorial risk communication of asymptomatic atherosclerosis has the potential to improve CVD prevention. It is therefore of importance to identify the active components of the intervention and the mechanisms of action. First of all, through pictorial presentation of atherosclerosis, the CVD risk was *conceptualized,* which might be of importance for understanding the link between lifestyle and the build-up of plaque and narrowing arteries. Since the ultrasound examination assessed the underlying process of CVD, the risk communication represents *feedback from one´s own body*, and the communication of risk is thereby also *personalized*. Our results show that the result letter was *easy to understand*, and according to our qualitative study, the message was perceived as *accurate and reliable*. The fact that *risk is related to age* also means that *consequences* of atherosclerosis are shown. The motivational conversation with the nurse aimed at strengthening *self-efficacy* and *response efficacy* to increase motivation for a healthy lifestyle and to empower the study participants. All of the above-mentioned components and mechanisms are in line with findings of a review assessing the effect of visual interventions on illness beliefs and medication adherence in chronic conditions. According to the review, the most common behavioural change techniques [51] identified for interventions with a sustained effect on adherence at post-intervention follow-up were *Information about health consequences, Salience of consequences*, *Credible source* and *Biofeedback* [10].

Regarding mechanisms of actions, our results show how important cognitive and emotional reactions are. This is in line with our qualitative findings, suggesting that an interaction between cognitive and emotional reactions is critical from early reactions to risk messages, through the decision-making process of behavioural change to maintenance of a healthy lifestyle. Theories of fear appeals typically assume that threat and efficacy interact to produce danger control (self-protective attitudes, intentions, behaviours) or fear control actions (defensive avoidance, denial, reactance). The *Extended parallel process model* suggests that threat (and corresponding fear) motivates a response, and that efficacy determines the nature of that response (either danger or fear control) [23]. The combination of high threat/high efficacy has been found to be most persuasive, whereas low threat/high efficacy and low threat/low efficacy are least persuasive [52]. Our findings do in part challenge this, since arousal in itself was associated to change in lifestyle index, and the fact that the combination of high cognitive response and high arousal was most beneficial. In other words: being worried does not seem to be a prerequisite for behavioural change. Even though negative emotions and cognitive response in combination yielded a similar result (not presented), arousal was more beneficial than negative emotions for change in lifestyle index, in line with analysis on separate emotional factors. These findings might indicate that the intervention has the potential to evoke not only a *disease prevention focus*, characterized by avoiding losses and taking precautionary actions, but also a *health promotion focus,* concerned with aspirations to preserve good health.

The correspondence between severity of atherosclerosis, as presented by the pictorial risk message, and cognitive and emotional responses, was perhaps somewhat weaker than expected. A possible explanation is that even a less severe risk message conceptualizes atherosclerosis as underlying process of CVD, and thereby raises awareness of the link between lifestyle and CVD. From an epidemiological perspective, and due to the great need of effective CVD prevention, this is of course very important. Since most cases of disease and deaths occur outside the high-risk group, risk communication strategies need to be effective not only for high-risk groups, but for the entire population. Furthermore, from the perspective of equality, an important finding is that increased understanding of individual CVD risk, and increased understanding of the possibility to influence risk and increased understanding on how to influence risk were more pronounced among participants without high education.

The strengths of the study include the long follow-up time regarding lifestyle factors, the population-based sample with asymptomatic atherosclerosis and a fairly good sample size. A limitation is that the lifestyle index was based on self-reported data. On the other hand, previously published studies within VIPVIZA show improvement in risk scores (representing objective measures) for the intervention group [30, 38], which implies that we have reason to trust the self-reported measures. Our qualitative study indicated that, among informants, the intervention made a strong and long-lasting impression. With the baseline ultrasound result as a starting point, reactions, beliefs and standpoints were evolving over time, where reactions in early stages laid the foundation for later stages of health-related decision making, behavioural change, awareness of disease risk and a changed mindset regarding food choice and physical exercise [50]. Still, a possible limitation in the present study is that the assessment of reactions was made in retrospect, three years after the first ultrasound letter. However, participants received several reminders, and the fact that we see a sustained effect on lifestyle modification might therefore indicate that *if* people do forget their reactions, the shown associations between cognitive and emotional reactions might actually be even stronger. Future research should consider separate analyses for the sexes and different education levels. In coming studies, we plan to assess risk perception and efficacy beliefs with other measures in a larger sample.

## Conclusions

This study demonstrates the potential of communicating asymptomatic atherosclerosis with a pictorial, colour coded and age-based strategy, also including a motivational conversation. Our results show the importance of CVD risk communication evoking engagement, and that interactions between cognitive and emotional reactions are central for sustained lifestyle modification. Furthermore, our results indicate that, in an asymptomatic population, atherosclerosis screening may strengthen both disease prevention and health promotion. The complexity of the results show that more research is needed, e.g. regarding how to assess and support a successful balance between emotional reactions and efficacy beliefs in patient consultation. Further research is also needed regarding the potential of pictorial presentation of atherosclerosis to target people with different education levels, and whether emotional engagement, rather than fear appeals in combination with efficacy beliefs, are important for behavioural change. Finding ways to promote health behaviour by supporting accurate risk perception and strengthening efficacy beliefs while taking emotional reactions into consideration should be a future assignment in research and clinical settings.

### Supplementary Information

**Additional file 1.** Overview of the study design.

**Additional file 2.** Source file containing BCTs from the Behaviour Change Intervention Ontology.

## Data Availability

The data sets used and analysed during the current study are available from the principal investigator of VIPVIZA, Professor Ulf Näslund, on reasonable request.

## Abbreviations

CVD: Cardiovascular disease
VIPVIZA: Visualization of Asymptomatic Atherosclerotic Disease for Optimum Cardiovascular Prevention
PROBE: Pragmatic, open-label, randomized controlled trial with masked evaluators
VIP: Västerbotten Intervention Program
IMT: Intima media thickness
