# Supplementary material for: From risk communication about asymptomatic atherosclerosis to cognitive and emotional reactions and lifestyle modification
Source: BMC Psychol. 2024 Jan 24;12:47. doi: 10.1186/s40359-023-01467-x (PMC10809670; doi:10.1186/s40359-023-01467-x)
Supplement: Supplementary file 1 — Additional file 1. Overview of the study design. [file 40359_2023_1467_MOESM1_ESM.pdf]

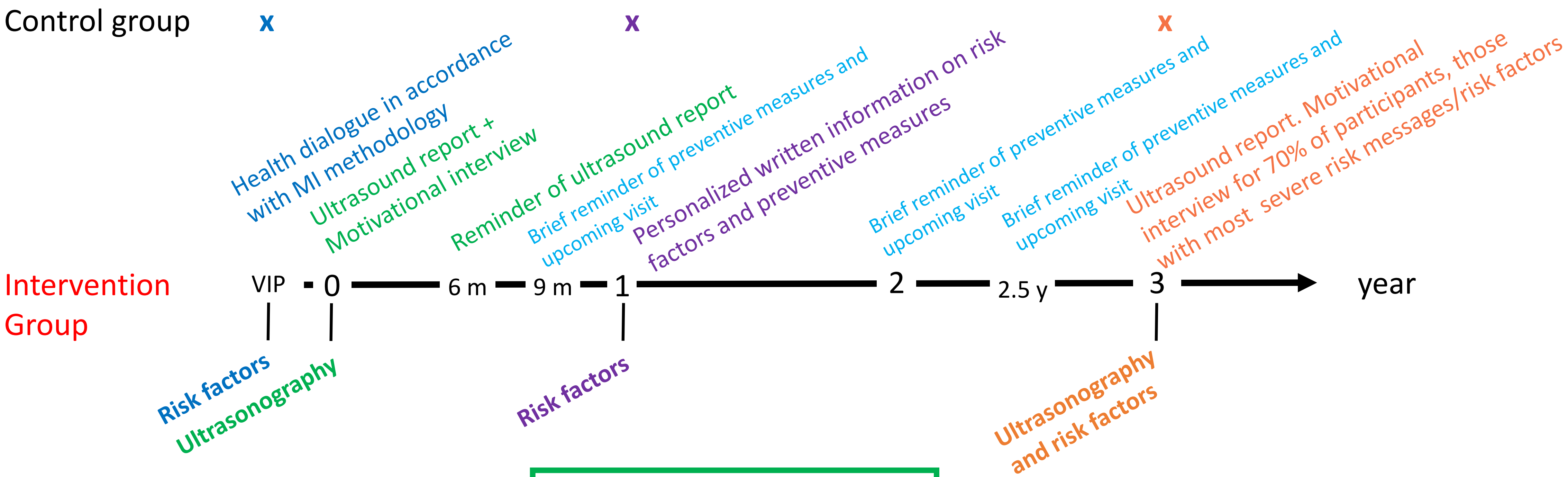

VIP:

- Attend to discrepancy between current behaviour and goal BCT BCIO:007012
- Provide feedback on outcome of behaviour BCT BCIO:007027
- Instruct how to perform behaviour BCT BCIO:007058
- Present information from credible influence BCT BCIO:007075
- Provide pharmacological support BCT BCIO:007145 (after VIP *if* referred to GP)

VIPVIZA baseline:

- Provide biofeedback BCT BCIO:007026
- Provide feedback on outcome of behaviour BCT BCIO:007027
- Instruct how to perform behaviour BCT BCIO:007058
- Inform about health consequences BCT BCIO:007063
- Increase salience of consequences BCT BCIO:007068
- Prompt intended action BCT BCIO:007080
- Present information from credible influence BCT BCIO:007075
- Persuade about personal capability BCT BCIO:007137

1-year follow-up:

- Provide feedback on outcome of behaviour BCT BCIO:007027
- Instruct how to perform behaviour BCT BCIO:007058

Additional file 1. Overview of the study design
